# Technology-Based Applications for the Assessment and Management of Alzheimer’s Disease and Related Disorders

**DOI:** 10.1093/geroni/igaa057.2905

**Published:** 2020-12-16

**Authors:** Lauren Massimo, George Demiris

## Abstract

Over the last decade, technological advances have made it possible for people to access health information right at their fingertips. Indeed, technology-based applications (apps) have been developed to tackle a wide range of health-related issues, including Alzheimer’s disease and related disorders (ADRD). As such, there is a critical need to develop and test effective dementia apps that can ultimately be converted in to scalable programs. In this interdisciplinary symposium, we will discuss the development and testing of novel apps for the assessment and management of ADRD. The first session will discuss the development of an online money management credit card task to assess cognitively vulnerable older adults who are at risk for making poor financial decisions. The second session will describe the development and psychometric testing properties of a mobile app to detect the earliest features of preclinical Alzheimer’s disease. The third session will highlight findings from a study testing a smartphone-based prompting app to improve everyday task completion in persons with mild cognitive impairment and mild dementia. The last session will share findings from a study testing a mobile app to increase goal-directed behavior and reduce apathy in persons with dementia. Together, these presentations describe how technology-based applications can be used to assess and manage cognitive and behavioral symptoms of ADRD.

